# A Physical Model Suggests That Hip-Localized Balance Sense in Birds Improves State Estimation in Perching: Implications for Bipedal Robots

**DOI:** 10.3389/frobt.2018.00038

**Published:** 2018-04-04

**Authors:** Darío Urbina-Meléndez, Kian Jalaleddini, Monica A. Daley, Francisco J. Valero-Cuevas

**Affiliations:** ^1^Department of Biomedical Engineering, University of Southern California, Los Angeles, CA, United States; ^2^School of Engineering, National Autonomous University of Mexico, Mexico City, Mexico; ^3^Division of Biokinesiology and Physical Therapy, University of Southern California, Los Angeles, CA, United States; ^4^Comparative Biomedical Sciences, Royal Veterinary College, London, United Kingdom

**Keywords:** balance, lumbosacral organ, vestibular system, birds, perch, compliant robot, co-localized sensing, distributed sensing

## Abstract

In addition to a vestibular system, birds uniquely have a balance-sensing organ within the pelvis, called the lumbosacral organ (LSO). The LSO is well developed in terrestrial birds, possibly to facilitate balance control in perching and terrestrial locomotion. No previous studies have quantified the functional benefits of the LSO for balance. We suggest two main benefits of hip-localized balance sense: reduced sensorimotor delay and improved estimation of foot-ground acceleration. We used system identification to test the hypothesis that hip-localized balance sense improves estimates of foot acceleration compared to a head-localized sense, due to closer proximity to the feet. We built a physical model of a standing guinea fowl perched on a platform, and used 3D accelerometers at the hip and head to replicate balance sense by the LSO and vestibular systems. The horizontal platform was attached to the end effector of a 6 DOF robotic arm, allowing us to apply perturbations to the platform analogous to motions of a compliant branch. We also compared state estimation between models with low and high neck stiffness. Cross-correlations revealed that foot-to-hip sensing delays were shorter than foot-to-head, as expected. We used multi-variable output error state-space (MOESP) system identification to estimate foot-ground acceleration as a function of hip- and head-localized sensing, individually and combined. Hip-localized sensors alone provided the best state estimates, which were not improved when fused with head-localized sensors. However, estimates from head-localized sensors improved with higher neck stiffness. Our findings support the hypothesis that hip-localized balance sense improves the speed and accuracy of foot state estimation compared to head-localized sense. The findings also suggest a role of neck muscles for active sensing for balance control: increased neck stiffness through muscle co-contraction can improve the utility of vestibular signals. Our engineering approach provides, to our knowledge, the first quantitative evidence for functional benefits of the LSO balance sense in birds. The findings support notions of control modularity in birds, with preferential vestibular sense for head stability and gaze, and LSO for body balance control,respectively. The findings also suggest advantages for distributed and active sensing for agile locomotion in compliant bipedal robots.

## 1. Introduction

All terrestrial vertebrates have linear and angular acceleration sense localized to the vestibular system of the inner ear. It is well-known that birds use a variety of reflexes mediated by internal signals to stabilize their head during walking and flying (Maurice et al., 2006). Uniquely among living animals, birds appear to have two specialized balance-sensing organs: the vestibular system of the inner ear and an additional balance sensor located between the hips called the lumbosacral organ (LSO) (Necker, 2006) which has been proposed to be especially useful for terrestrial locomotion (Necker, 2005, 2006). Birds have long flexible necks, with head motions tightly coupled to gaze control (Necker, 2007; McArthur and Dickman, 2011; Pete et al., 2015). Consequently, the vestibular system is not closely nor tightly coupled to the torso. In contrast, the LSO is located in the sacrum between the hips, near the CoM. Having a balance organ at the torso is likely to be beneficial to legged locomotion and balance because the hip joint plays an important role on controlling the position of the CoM of the whole body with respect to the foot (Abourachid et al., 2011). Here we consider and contrast the functional implications of hip-localized (LSO) and head-localized (vestibular) balance-sense.

Generally speaking, keeping balance is a task that many legged-animals perform to prevent falling or rotating about the foot point after perturbations (Vukobratovic et al., 2012). Specifically, a balance-sensing organ produces afferent signals to detect current body posture and motion to determine the movements required to achieve or maintain a desired posture and motion. In birds, direct neurophysiological evidence has clearly established that they must possess balance sense that is independent of the vestibular system (Abourachid et al., 2011). They retain the ability to reflexively compensate for body rotations even after labyrinthectomy and spinal cord transection to eliminate descending inputs influenced by the vision and vestibular senses (Abourachid et al., 2011). This neurophysiological evidence, along with particular anatomical features of avian lumbosacral region (below), suggests a balance sensing function of the LSO that complements proprioceptive information from the vestibular system, as well as mechanoreceptors in the skin, joints and muscles.

Anatomically, the LSO is located within an enlargement of the lumbosacral region of the spinal column, between the 27 and 38th segments (Streeter, 1904). The LSO presents a suite of features unique to the spinal column of birds, including bilateral protrusions of neural tissue identified as mechanosensors (accessory lobe (AL) neurons), located adjacent to ligaments supporting the spinal cord (Schroeder and Murray, 1987; Necker, 2005, 2006; Yamanaka et al., 2008). The spinal cord is dorsally bifurcated in this region and supports a “glycogen body” (GB) centered on top. The entire region is enclosed by bony canals with a distinct concentric ring structure (Necker, 2006). The arrangement of the canals, AL, ligaments, and GB is reminiscent of the vestibular system (Necker, 2006) and invites functional analogy to an accelerometer. Each AL contains mechanoreceptors (Schroeder and Murray, 1987; Necker, 2006; Yamanaka et al., 2008), with commissural axons projecting to last-order premotor interneurons in the spinal pattern generating network (Eide and Glover, 1996; Necker, 2006). The AL neurons within the LSO exhibit spontaneous firing and phase-coupled firing in response to vibrational stimulation between 75 and 100 Hz, and ablation of these neurons disrupts standing balance (Necker, 2006). Thus, multiple lines of anatomical and neurophysiological evidence suggest balance-sensing function of the LSO.

Despite evidence of LSO hip-localized balance-sense in birds, no previous studies have provided quantitative evidence for the functional benefits of LSO as an adaptation for posture balance sensing of posture-relevant information. We hypothesize that hip-localized balance sense provides two main functional advantages compared to head-only balance-sense: (1) reduced sensorimotor delay and (2) more accurate state estimation of foot-ground acceleration due to closer proximity to the feet. Here we use a physical model of a perching guinea fowl subject to foot-ground perturbations to test the hypothesis that hip-localized balance sense enables more rapid sensing and accurate state estimation compared to only a head-localized balance sense.

Most birds “perch” (balance with the feet attached to the substrate) when they alight on elevated objects such as branches; therefore we focus on perching as a conveniently simple and ecologically relevant balancing behavior. We built a simple physical model of a standing guinea fowl perched on a horizontal platform (i.e., feet attached to the platform). The horizontal platform was attached to the end effector of a 6 DOF robotic arm, allowing us to apply perturbations analogous to motions of a compliant branch. The physical model provides a first approximation of the muscle-tendon viscoelastic properties that provide leg compliance. We approximated LSO and vestibular balance sensors using 3-D accelerometers located at hips and at the head, respectively. We used system identification to estimate foot-ground acceleration as a function of hip- and head-localized sensing, individually and combined.

## 2. Materials and methods

### 2.1. Physical model of a guinea fowl

**Figure 1:**
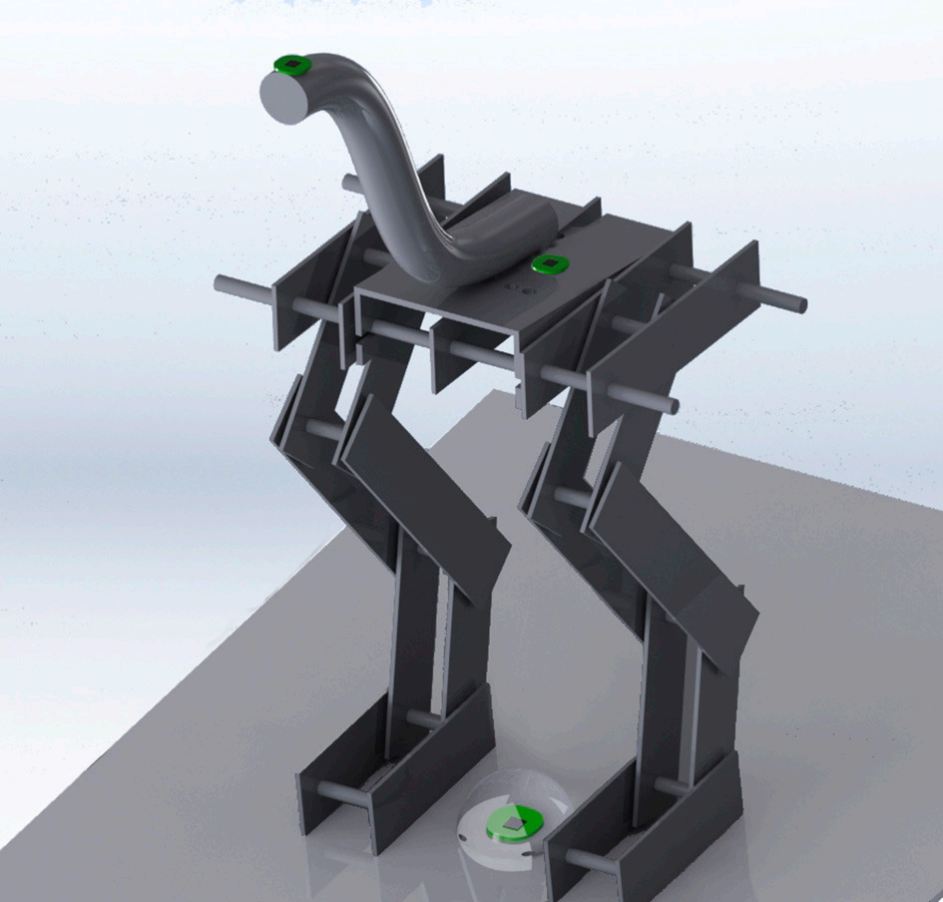
Physical model of the skeleton of the guinea fowl made of articulated aluminum plates and an elastic tube for a neck. The location of the sensors can be seen on the floor between the model's feet, on its pelvis between the hips, and on its head. The joints of the model are, starting from the pelvis: the hip, knee, ankle and metatarsal joints. The transparent sphere around the accelerometer between the feet indicates the scale of random displacements 20 mm in radius.

**Figure 2:**
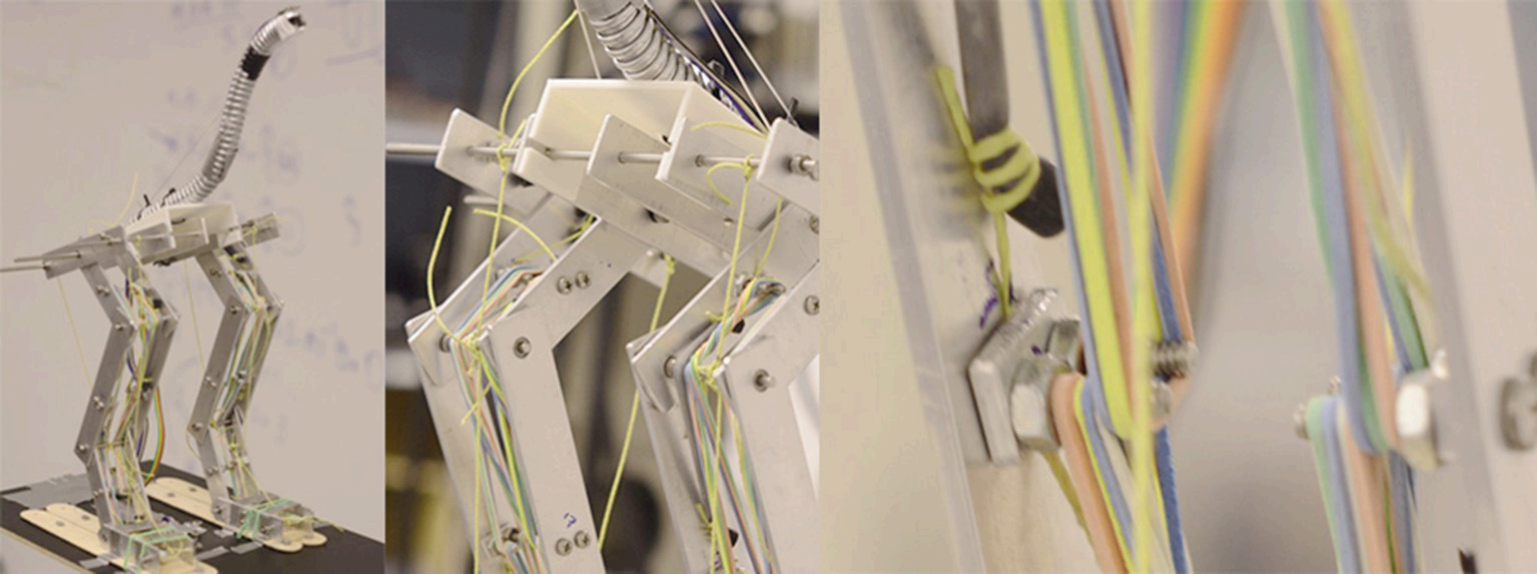
Photographs of the physical model of the skeleton of the guinea fowl. On the left the complete model is shown, on the middle and right sections details of the elastic linkages that are required for the robot to maintain a standing posture can be seen.

A skeletal model of a guinea fowl was built by interconnected and hinged aluminum bars (Figures 1, 2). Friction was reduced by using bearings at the hip, knee, ankle, and foot. The general body size, limb segment lengths and configuration were based on guinea fowl anatomy from the literature (Gatesy and Biewener, 1991; Daley et al., 2007; Gordon et al., 2015), with a hip height of 20 cm.

This physical model focused on approximating the guinea fowl's (i) LSO (hip) and vestibular (head) balance sensing systems location, (ii) body center of mass location and limb configuration in a standing posture and (iii) visco-elastic mechanical properties of the muscle-tendon-driven limbs. This model was meant as a first approximation of the key physical features, to allow a quantitative comparison of the information available at hip- and head-localized balance sensors. It was not meant to be an exhaustive exploration of the effects of posture, material properties, and muscle-tendon actions. Such considerations could be the subject of future work.

The toes of the model were firmly attached to a platform. Thus, the guinea fowl model “perched” while maintaining an upright standing posture. This posture was maintained by the passive tensions in rubber bands that cross the hip, knee, ankle and metatarsal joints without further assistance or active support (Figure 2). We pre-tensioned rubber bands across joints to represent the tendon-driven functional anatomy of a guinea fowl. These rubber bands also have viscoelastic properties that approximate the passive mechanical properties of “muscles” held at a constant activation level when holding the standing posture. The origins and insertions of the rubber bands were adjusted to have large enough moment arms at each joint to overcome gravity and maintain posture even when perturbed by the moving platform. Our focus was not to explore effects of varying muscle activation patterns for standing postures, but instead to find a set of tensions in the rubber bands sufficient to maintain standing posture and propagate the perturbations from the platform through the skeletal anatomy.

We used two interchangeable necks, each with different stiffness to test the effects of muscle coactivation on balance sensing at the head. Each neck was 25 cm long and curved as shown in Figures 1, 2. The first neck was made of 12.7 mm diameter Ultra-Flex Corrugated Steel Sleeving (McMaster-Carr, part 54885K21). The second was 19.05 mm diameter Abrasion-Resistant Polyurethane Rubber Rod (McMaster-Carr, part 8695K155).

### 2.2. Instrumentation

**Figure 3:**
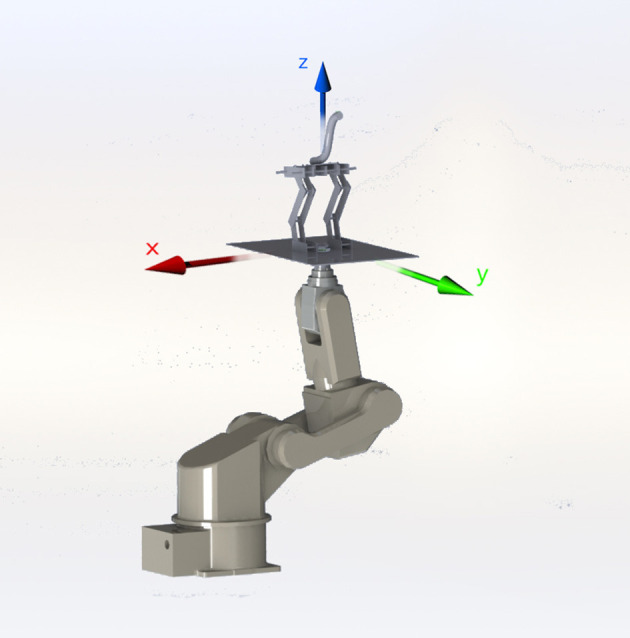
Generating 3D movements with the 6-DOF AdeptSix 300 robotic arm enabled us to apply repeatable and specific type of perturbations to our model.

The end-effector of a 6 degrees of freedom (DOF) AdeptSix 300 robotic arm (Omron Adept Technologies, Inc, San Ramón, CA) hold the platform where the model perched (Figure 3). We used 3-D accelerometers at the following locations on the model: (i) head to represent the vestibular system; (ii) hip to represent the LSO sensor, and (iii) between the feet to record the reference perturbations or “foot acceleration” (Figure 1). All accelerometers were MEMS inertial sensors Model LIS344ALH (ST Microelectronics, Geneva, Switzerland).

### 2.3. Trials

Each trial replicated a scenario that a guinea fowl might face while perching on a tree branch which is subject to perturbations from weather and other animals. Our goal was not to replicate natural perturbation exactly, but to provide a general test of our hypothesis that the LSO has benefits over the vestibular system for rapid and accurate state estimation for balance.

Each trial consisted of a series of 3,000 random, uncorrelated displacements generated by the robotic arm. Each displacement was a center-out-and-back movement in a random direction to the surface of spheres with 2, 5, 10, and 20 mm in radius. Trials were block-randomized across sphere sizes. We recorded a total of eight trials (4 sphere sizes × 2 necks stiffnesses) (Table 1).

**Table 1:** Each trial consisted of 3,000 random center-out-and-back displacements (center-surface of a sphere).

**Trials**	**Low stiffness neck**	**High stiffness neck**
2 mm sphere	*Trial_LS_2_*	*Trial_HS_2_*
5 mm sphere	*Trial_LS_5_*	*Trial_HS_5_*
10 mm sphere	*Trial_LS_10_*	*Trial_HS_10_*
20 mm sphere	*Trial_LS_20_*	*Trial_HS_20_*

### 2.4. Data acquisition

We used a high-performance National Instruments (NI) *PXI-8108* computer, upgraded with 4 GB DDR2 RAM and a 500 GB SSD. An NI *PXI-6254* ADC card recorded the accelerations signals. The data acquisition hardware was housed in the NI *PXI-1042* chassis. We acquired data at the sampling rate of 1 kHz.

### 2.5. Data analysis

#### 2.5.1. Estimation of neck stiffness

To estimate the effective neck stiffness, we performed a boot-strap analysis of 1,000 trials by randomly selecting 30 s segments from each trial. We then found the resonant frequency (the frequency with maximal power) of accelerations at the head. The effective muscle stiffness was estimated from:

(1) $$\begin{array}{r} K_{i}=m_{i}f_{i}^{2} \end{array}$$

where *i* is the neck number, *m*_*i*_ the mass and *f*_*i*_ the resonant frequency.

#### 2.5.2. Estimation of sensory delay at the hip and head

We calculated cross-correlation of foot acceleration against that recorded from hip or head to estimate the propagation delays of the applied mechanical perturbations. The delay was taken as the lag where the cross-correlation was maximal.

#### 2.5.3. Estimation of the time history of foot acceleration

We used a data-driven modeling approach to estimate the time history of the foot acceleration given the time history of signals recorded at the sensory sites (hip and head). To this end, we trained state-space models (in the least-squares sense) to predict foot acceleration from the hip or head accelerations. We used MOESP state-space identification (Verhaegen and Dewilde, 1992; Verhaegen and Verdult, 2007) implemented in the *State-space Model Identification* (SMI) MATLAB toolbox (Haverkamp and Verhaegen, 1997). The state-space model is represented as follows:

(2) $$\begin{array}{r} \left\{ \begin{array}{cc} x\left(k+1\right)\; & =Ax\left(k\right)+Bacc_{\text{sensor}}\left(k\right)\\ acc_{\text{foot}}\left(k\right)\; & =Cx\left(k\right)+Dacc_{\text{sensor}}\left(k\right) \end{array}\right. \end{array}$$

where *acc*_sensor_(*k*) is the input signal (acceleration signal recorded from the hip or neck) and *acc*_foot_(*k*) is the measured foot acceleration. *x*(*k*) is the state variable, and *A*, *B*, *C*, *D* are the unknown state-space matrices. We set the model order to three after inspecting the singular values of the extended observability matrix as described in the previous work (Haverkamp, 2000). The model order of three resulted in 21 parameters that was significantly less than the number of 4,000 available training data points for each training run. Since the number of free parameters was much <10% of the training data, the model is not over-parameterized and cannot learn noise and the stochastic behavior.

We assessed the performance of the model in predicting the foot acceleration (${\hat{acc}}_{\text{foot}}$). By running the identified models in the prediction mode, we compared the predictions to the actual measured signals, *acc*_foot_. We quantified the difference using the identification *Variance Accounted For* (VAF) expressed as:

(3) $$\begin{array}{r} \%\text{VAF}=100\left(1-\frac{var\left({\hat{acc}}_{\text{foot}}\left(k\right)-{acc}_{\text{foot}}\left(k\right)\right)}{var\left({acc}_{\text{foot}}\left(k\right)\right)}\right) \end{array}$$

where 100% indicates a perfect prediction of all the variability in the measured signals, and 0% means no meaningful prediction.

#### 2.5.4. Boot-strap analysis and statistics

To estimate the robustness of the analyses (cross-correlation, system identification, etc.), we performed a 100 trials boot-strap study (random sampling with replacement) (Efron and Tibshirani, 1994). For each trial, we randomly chose 40 s windows from the measured data, performed the cross-correlation and system identification analyses, and then calculated summary statistics across the 100 measures. We performed student's *t*-test for statistically significant differences between conditions. Values for central tendency and variance are reported as medians (interquartile range) unless stated otherwise.

## 3. Results

We first present the differences in neck stiffness, then the effect of sensor location and neck stiffness on (i) sensing delay, and (ii) estimation of foot acceleration.

**Figure 4:**
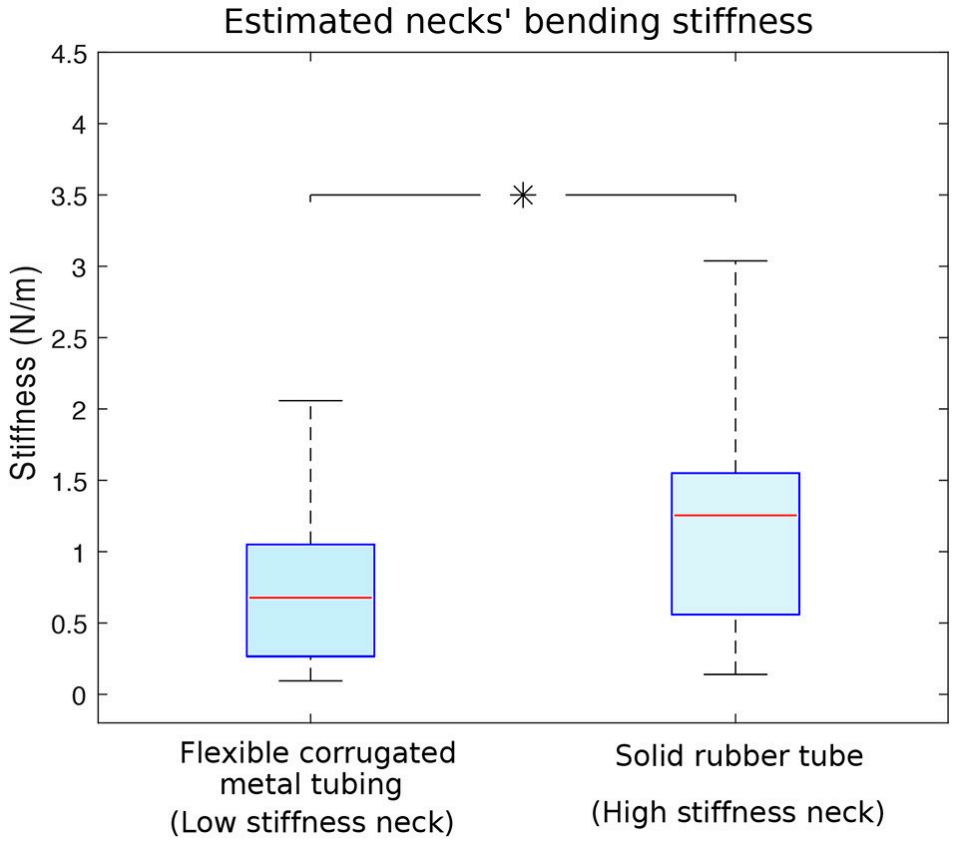
Estimated bending stiffness of the two necks. Neck stiffnesses were calculated using data from 1,000 different trials and the simple lumped-parameter model in Equation 1. **Left:** stiffness calculated for the flexible corrugated metal tubing (i.e., low stiffness neck). **Right:** stiffness calculated for the solid rubber tube (i.e., high stiffness neck) Flexible corrugated metal tubing and solid rubber tube data were statistically different (with *p* < 0.05 and indicated with an asterisk), their respective medians are 0.67 and 1.25 N/m.

The necks made from two different materials have different bending stiffnesses whose estimates are shown in Figure 4. Since we measured the dynamical response of the entire physical model (see section 4), each direction and magnitude of perturbation induced a different dynamical response that resulted in a different acceleration measured at the head. This led to different resonant frequencies to be multiplied by the mass of the neck (Equation 1). Note that we would obtain different estimates of neck stiffnesses if the square of the resonant frequency at the head were multiplied by the mass of the whole model. Doing this would have given us an approximation of the stiffness of the whole body, which besides the neck, has a fixed stiffness. Also, if the complete body mass were considered, mass differences between trials with different necks would have been smaller, resulting in a constant bias that would not change the statistical differences between the estimates of neck stiffness. The median neck stiffnesses were 0.67 (0.26–1.05) N/m and 1.25 (0.56–1.55) N/m for the low and high stiffness necks, respectively. Student *t*-test shows the average neck stiffness are significantly different (*p* < 0.05).

**Figure 5:**
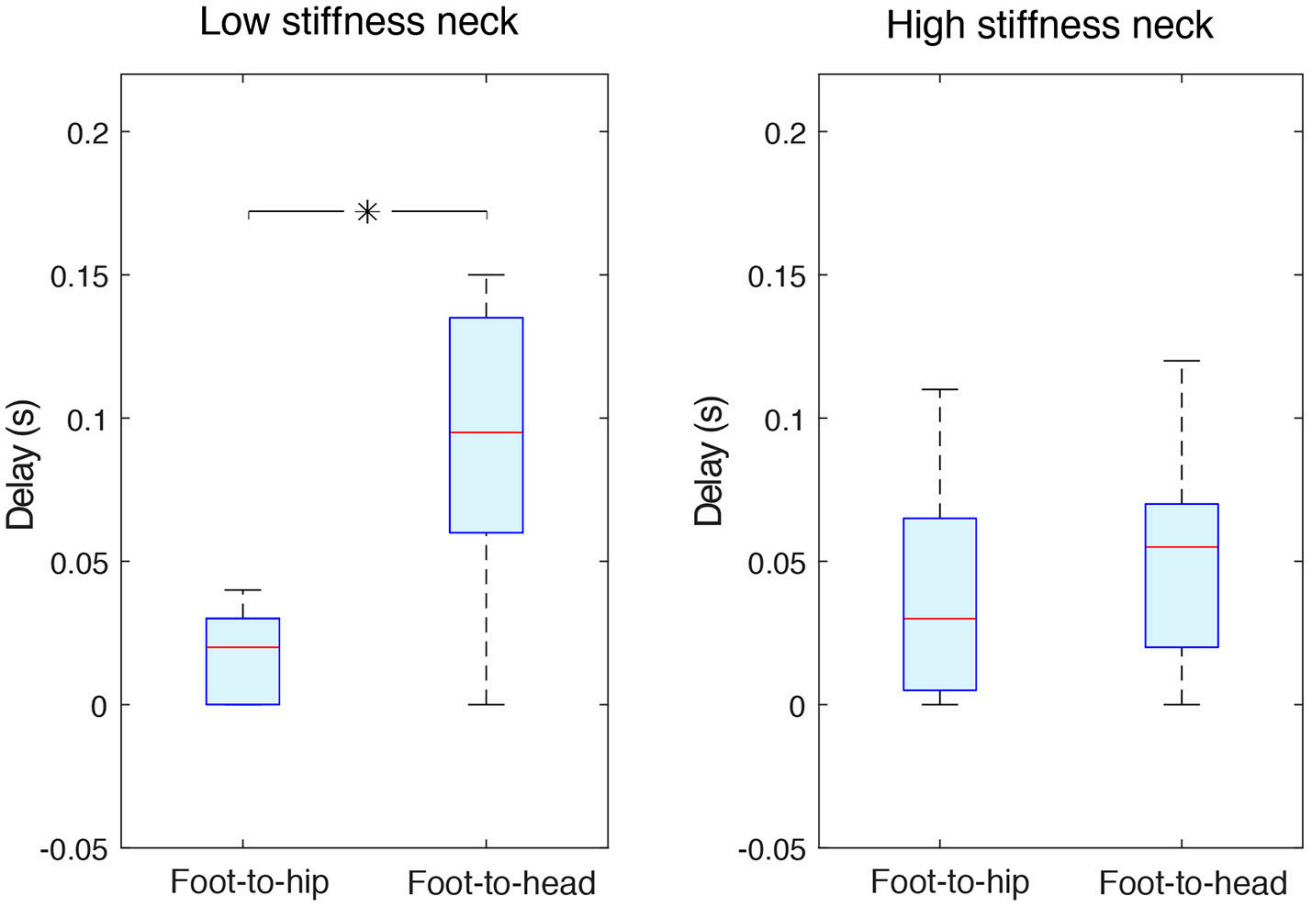
Independently of the neck stiffness, foot-to-hip delays were shorter than foot-to-head ones. The two data groups in the panel corresponding to the low stiffness neck **(left panel)** were statistically different (with *p* < 0.05 and indicated with an asterisk); this is not the case for high stiffness neck data **(right panel)**. Foot-to-head median delays were longer, measured at 0.095 s for the low stiffness neck, and 0.055 s for the high stiffness one. Foot-to-hip median delays were 0.02 and 0.03 s respectively for the low stiffness and high stiffness necks.

As expected, an accelerometer at the hip generally detected foot acceleration with shorter delays than the accelerometer at the head. Foot-to-hip median delays were 0.02 (0–0.03) s and 0.03 (0.005–0.065) s, respectively for the low and high stiffness necks. Foot-to-head median delays were longer, measured at 0.095 (0.06–0.135) s for the low stiffness neck, and 0.055 (0.02–0.07) s for the high stiffness one (Figure 5). The variability was quite large as the shown information collapses data across different acceleration axes and different sphere experiments (perturbation magnitudes).

Foot-to-hip delays were significantly shorter than foot-to-head delays (*p* < 0.05) for the low stiffness neck, but not significantly different for the high stiffness neck (Figure 5). A stiffer neck reduced the delays for information sensed at the head. This resulted in hip and neck delays that were very similar with no statistical difference.

**Figure 6:**
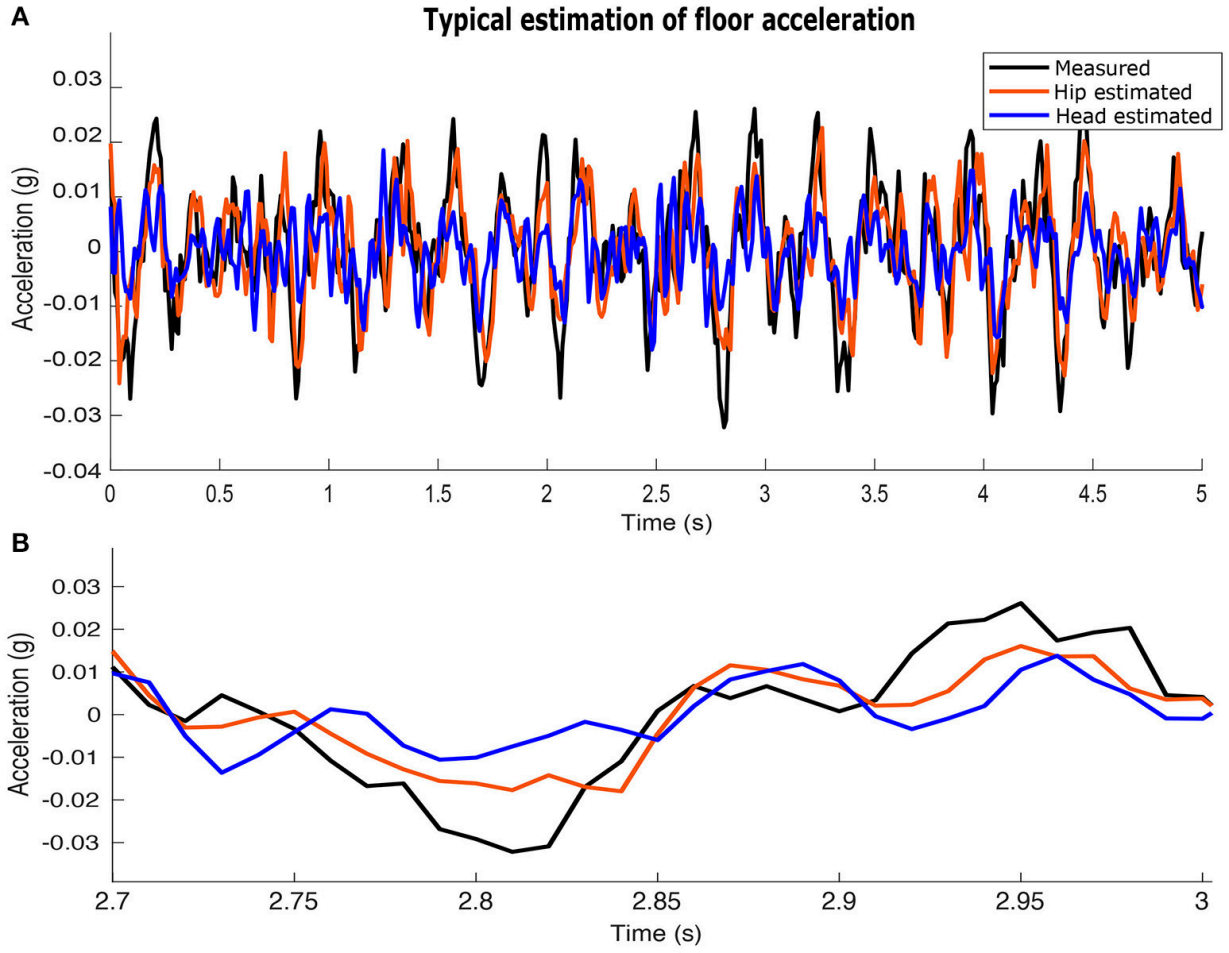
Example of the acceleration at the feet in the sagittal plane estimated from the measured accelerations at the hip and head. The acceleration at the hip yields more accurate estimates of acceleration at the feet. **(A)** a 5 (s) time window. **(B)** a 300 (ms) time window.

Estimates of acceleration at the feet are more accurate when using signals from the hip-mounted accelerometers than from the head-mounted accelerometers. Figure 6 shows an example where acceleration at the feet is estimated from the hip- and head- mounted accelerometer, overlaid with the ground-truth signal measured at the feet.

**Figure 7:**
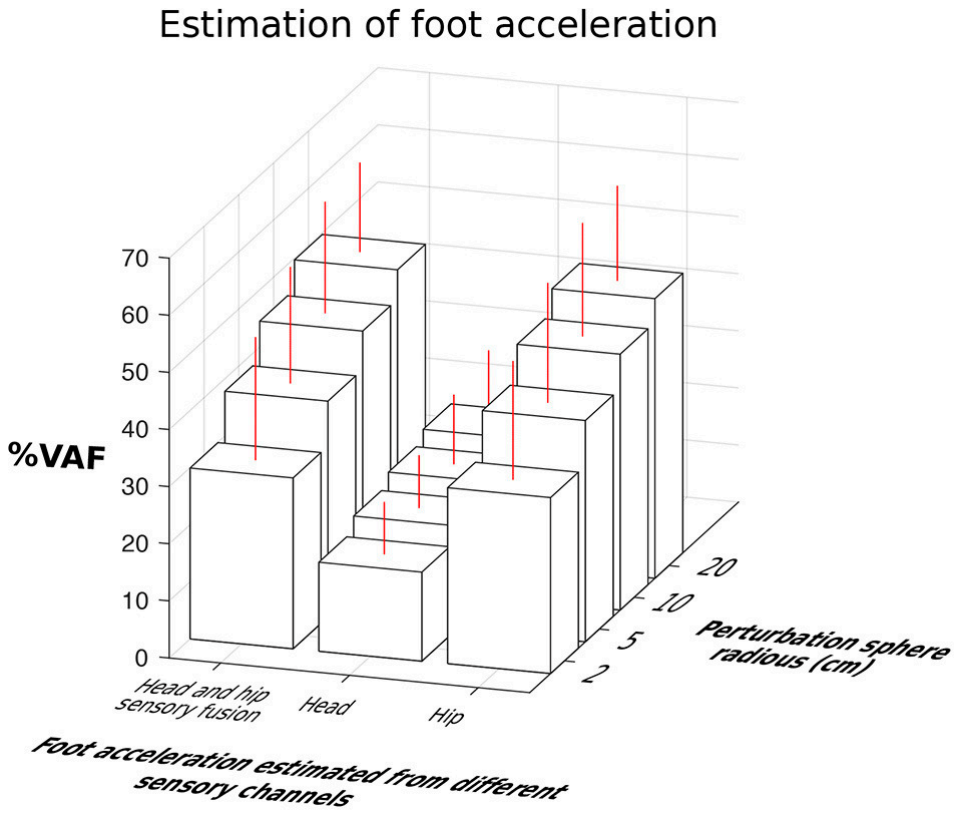
Comparison of estimation accuracy of foot acceleration from the hip, head and their fusion as a function of perturbation magnitudes. Hip-to-feet compared to head-to-foot acceleration estimation was more accurate (*p* < 0.05). Fusion of the hip and head information did not improve estimation of the foot acceleration beyond that obtained with hip information along.

**Figure 8:**
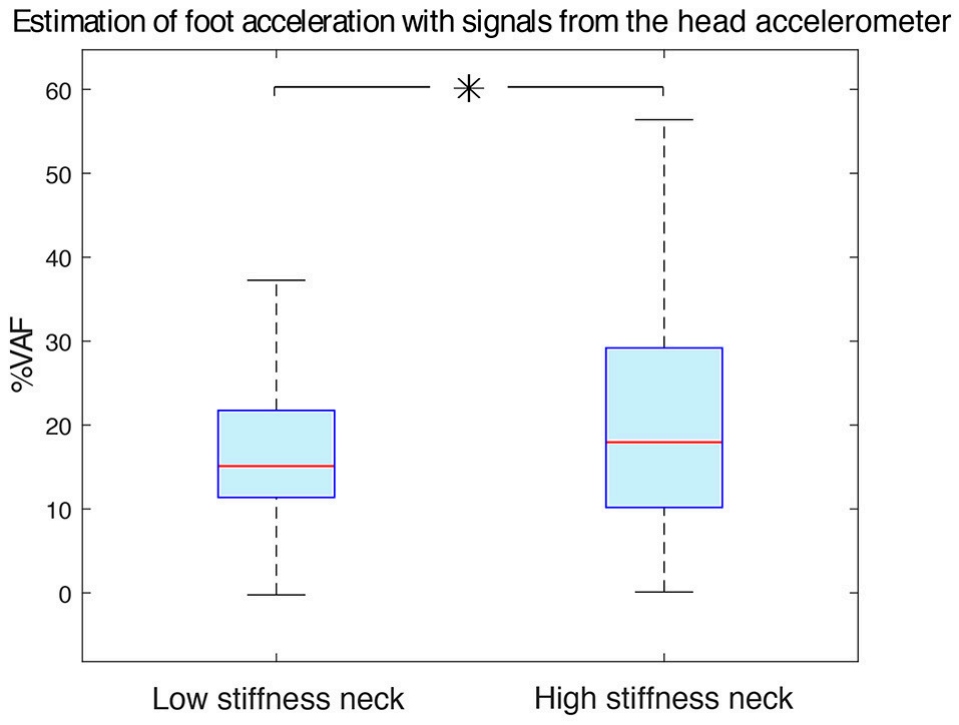
Estimation accuracy of foot acceleration as a function of neck type. Increasing neck stiffness improved estimation of foot acceleration from acceleration measured at the head. Low stiffness and high stiffness neck data were statistically different (with *p* < 0.05 and indicated with an asterisk). The median %VAF was 15.11 and 17.95 for the low stiffness neck and high stiffness neck respectively.

Hip-localized estimates of the foot acceleration accounted for 30.81–48.96% of variance (% VAF as defined in Equation 3) against 15.59–22.19% of head-localized estimates (Figure 7). This figure summarizes the estimation results by pooling together data from both neck stiffnesses. Prediction of foot acceleration as a function of neck type is shown in Figure 8. Particularly, Figure 7 shows data separated as a function of perturbation magnitude. It demonstrates that independently of the perturbation magnitude, the estimate of foot acceleration from the hip was always more accurate than that from the head sensor. Moreover, sensory fusion (combining info from both sensors) did not significantly improve the foot acceleration estimation. Therefore, sensory fusion did not provide additional benefits beyond hip-only sensing.

We have found that when only head-localized accelerometers were available, the high stiffness neck improved estimates of foot acceleration compared to the low stiffness neck (Figure 8). With the low stiffness neck, the median VAF was 15.11 (11.38–21.74)%, while it was 17.95 (10.18–29.19)% for the high stiffness one. These data groups were statistically different (*p* < 0.05).

## 4. Discussion

To validate the anatomical and neurophysiological evidence of LSO balance sensing function in birds, we present a quantitative investigation of the functional benefits of hip-localized balance sense. Here we investigated the perturbation sensing dynamics of a physical model of a guineafowl perched in a standing posture. We explored two proposed functional advantages of hip-localized compared to head-localized balance sense: minimization of sensorimotor delay and improved estimation of foot-ground acceleration, due to closer proximity of the sensor to the feet. To our knowledge, this is the first study to quantitatively analyze the practical benefits of hip-localized sensing of accelerating for balance control. We find that a hip-localized acceleration sensor—analogous to the LSO—provides shorter delays and improved state estimation of feet acceleration during substrate perturbations.

In particular, our experimental paradigm applied displacements at the feet, where we also measured the “ground truth” acceleration of the moving substrate on which the bird is perched. We then compared the ability to sense and reconstruct that ground truth acceleration on the basis of accelerations measured at the hip and head. We find that the location of these simulated balance sense organs has important consequences to how a bird (a model of a guinea fowl, in this case) could use acceleration information from hip-localized balance sense for bipedal perching, standing and locomotion. A second level of analysis focused on the material properties of the neck of the physical model. One was (less stiff) corrugated tubing, and the other (more stiff) solid rubber tubing. These material differences were designed to explore the effect of muscle co-contraction at the neck as a means of active sensing, or at least modulation of the utility of head-localized balance sense.

Before discussing the results in detail, it is important to clarify some features of our experimental approach to balance sense. A salient feature of our experimental results is the variability in our results, as in Figure 4. Shouldn't the bending stiffness of each neck be thought as a single number? Similarly, shouldn't the foot-to-hip delays be constant and the same independently of neck stiffness (Figure 5)? Recall that the stiffness of the system is inferred from the resonant frequency of the acceleration measured at the head. The acceleration at the head is a function of the the dynamical response *of the entire guinea fowl model* to input perturbations. In fact, we are measuring the frequency response and delays of the coupled oscillations of the legs held in a standing position by rubber bands, plus the pelvis and neck. Given that this physical structure is only symmetric in the sagittal plane, its dynamical response will depend on the direction of the 3D perturbations—which naturally results in variability in our results. Nevertheless, the corrugated tubing condition (“low stiffness neck”) leads to perturbation responses at the head that, in general and on average, reflect a lower stiffness for this lumped-parameter analysis. Similarly, foot-to-hip delays were, in general and on average, shorter than the feet-to-head delays. In a sense, instead of “neck stiffness,” the results in Figure 4 may be better called the “apparent stiffness lumped at the head.” But given that the purpose of this analysis is to test for the effect of the material properties of the neck on time delays and estimation accuracy, we chose not to belabor this point and simply call it “neck stiffness.” After all, (i) the neck is the only body part that was swapped, and (ii) changes in material properties only at the neck better reflect the potential effects of muscle co-contraction at the neck in the guinea fowl.

There are limitations to our approach that, while worth mentioning, we believe do not challenge the validity of our results. Importantly, our physical model can only approximate the anatomy and muscle mechanics of the guinea fowl. Our multi-link articulated structure approximates only the general link-segment arrangement and length proportions of the animal skeleton, and the viscoelastic rubber bands only roughly approximate the properties of muscle-tendon linkages. Similarly, we did not consider the proprioceptive signals coming from the joints, skin and muscles that could also contribute to state estimates of foot acceleration. While these limitations prevent us from claiming that our results are direct parallels of how a guinea fowl would respond neuro-mechanically to perturbations, it is nevertheless a valid means to *test for differences in sensory signals as a function of sensor location and neck stiffness*. Moreover, we explicitly avoided making the assumption that the skeleton of the guinea fowl was simply a set of links rigidly fused at a given posture. Rather, we used rotating hinges at the joints, where the posture of the model was achieved by appropriately setting the lengths and tensions of the rubber bands to approximate muscle-tendon actions to maintain posture at rest. This mechanical structure—as a first approximation—provides a biomechanically realistic instantaneous response to a perturbation at the feet, and avoids other multiple assumptions associated with a computational model (Martins et al., 2009). The results we present here are an analysis of the aggregate acceleration responses to a sequence of center-out 3-dimensional perturbations. As such, we consider the details of each response only implicitly. Future research could explore the moment-to-moment details of the responses within an individual perturbation.

The biological interpretation of these results hinges on the assumption that the functional benefits of hip-localized balance sense could translate into selective evolutionary pressure to promote the anatomical specialization of the LSO in evolutionary time. This assumption is supported by two fundamental control-theoretical notions: (i) that delays are detrimental because they make any causal closed-loop controller (biological or engineered) more unstable (Gu et al., 2003) and (ii) that having a more faithful estimate of a perturbation improves the corrective response, and thus improving performance, economy and stability.

The simplest interpretation of the time delays hinges on the notion that a causal feedback controller has knowledge of the past, but not of the present (strictly speaking) or future. Therefore, it cannot execute anticipatory control actions and is thus limited by its closed-loop bandwidth. In contrast, biological systems are well-known to produce anticipatory motor commands (Aruin and Latash, 1995; Westwick and Perreault, 2011), as well as short-latency reflexive responses (Sinkjær et al., 1999; Jalaleddini et al., 2017b,a). Anticipatory strategies are considered to be critical adaptations to mitigate the deleterious effects of large transmission and processing delays inherent to neural systems (Bean, 2007; Faisal et al., 2008). Nevertheless, any voluntary, anticipatory or reflexive action would benefit from shorter delays. This point is supported by the observation of many morphological and physiological adaptations in the nervous systems to reduce time delays such as increased axonal diameter, myelination and saltatory conduction.

The biological relevance of state estimation (Kalman, 1960; Simon, 2006) relates to the fact that physiological sensory signals contain task-relevant information, but not necessarily in the coordinates and units used by the controller. In particular, some version of the “state” of the system is encoded in sensory coordinates and units that are different from those used by the neural controller to select, plan and execute a response. This means that any raw sensory signal (e.g., acceleration at the LSO or vestibular system) must first be interpreted to extract useful information (e.g., the details of the perturbation at the feet). The MOESP state-space identification technique is but one example of a state estimator (Verhaegen and Dewilde, 1992; Verhaegen and Verdult, 2007). To test our hypothesis, it suffices to show that a hip-localized balance sensing organ is better at sensing, estimating, *and reconstructing* the perturbations at the feet than a head-localized one, Figure 6. On the same figure, we only show forward/backward accelerations (i.e., along the y axis), which are the most destabilizing during locomotion. It has been shown that lateral (i.e., side-to-side) movements are more stable than forward/backward movements because stance width naturally provides a stabilizing effect (Dean et al., 2007). Whether and how the concept of state estimation applies to the nervous system, however, is yet unresolved (Loeb, 2012).

Necker stated in the concluding paragraph of his 2006 paper that “The local organization of the neuronal network [of the LSO] favors rapid and hence effective control,” with no further elaboration (Necker, 2006). We now present what is, to the best of our knowledge, the first concrete evidence that a hip-localized balance sense organ (like the LSO) is an effective source of *faster* and *better* sensing of posture-relevant information. Faster sensing is evidenced by the shorter time delays for hip-localized vs. a head-localized accelerometers. Moreover, our results also show that the time delays for head-localized balance sense organs can be shortened by cocontracting neck muscles (i.e., a stiffer neck). From the state estimation point of view, however, we find that hip-localized balance sense organs are superior, and do not benefit from sensory fusion with head-localized acceleration—independently of neck stiffness. Therefore, we conclude that hip-localized balance sense indeed promotes more rapid and effective control.

These results have important implications for how the evolution of hip-localized balance sense by the LSO might have contributed to the unique sensorimotor control features of birds. In particular, it has long been recognized that birds have relatively “modular” function and control of wings, legs and tail compared to other vertebrates (Gatesy and Dial, 1996). The functional dissociation between forelimb (wing) for aerial locomotion and hindlimb (leg) for terrestrial locomotion is paralleled by increased autonomy of their respective sensorimotor control networks (Biederman-Thorson and Thorson, 1973; Jacobson and Hollyday, 1982; Sholomenko and Steeves, 1987; Ho and O'Donovan, 1993; McArthur and Dickman, 2011). The presence of a local and distributed balance sensing organ that is directly integrated with hindlimb spinal networks has likely contributed to this modular control organization. The mechanosensing neurons of the LSO project directly to pre-motor neurons in the spinal cord (Eide, 1996; Necker, 2006). This suggests the balance sense information produced by the LSO is likely to contribute to rapid and effective control because it is processed locally. Such local processing is advantageous because involving the brain in the response could introduce counterproductive time delays.

While our results focus on perching, hip-localized balance sense is likely beneficial for other postural and locomotor tasks. We designed our perturbations to simulate sensory inputs analogous to bird perching on a branch subject to varied 3-D movements such as wind, movements of other animals, etc. During perching, a bird is exposed to 3-D substrate perturbations, for which short-latency reflex responses could suffice, if sufficiently rapid sensing is available. This is similar to the observed knee and ankle strategies in the control of human upright posture (Bingham et al., 2011), or slip-grip mechanisms for human finger manipulation (Cole and Abbs, 1988). Moreover, such rapid and informative sensing is also critical to low-level (distributed, spinal or sub-cortical) sensorimotor processing to control short-latency responses to perturbations (Lawrence et al., 2015a,b) that ultimately supports long-latency control of voluntary function in general. The LSO is directly integrated with the hindlimb spinal motor control networks (Eide, 1996; Necker, 2006), suggesting that hip-localized balance sense is likely relevant to all hindlimb-mediated behaviors, including perching, standing balance, over-ground locomotion and arboreal locomotion. Birds effectively have two distinct balance sensorimotor processing centers: the “cerebral brain,” responsible for executive function and navigation, and the “sacral brain,” responsible for low-level, short latency control of terrestrial perching, standing and locomotion.

Adopting lessons from the millions of years of biological evolution poses intriguing and exciting possibilities for the *engineering* evolution of robust and versatile bipedal robots. There are well-known forms of morphological control where the structure of the body co-evolves with the nervous system (or controller) to simplify and improve open- or closed-loop control (Lipson and Pollack, 2000; Valero-Cuevas et al., 2007; Pete et al., 2015). At the other extreme we have the classical robotics approach to fully centralized control that depends on algorithms that process sensory information and issue motor commands. The LSO provides support for an intermediate alternative, where one can have the benefits of morphological adaptations and central control—but supplemented by distributed neural control centers informed by distributed balance sense organs like the LSO.

## Author contributions

DU-M designed and constructed the physical model of the guinea fowl, wrote the Title, Abstract, Introduction, did renders of the physical model of the guinea fowl and put together different author ideas and perspectives. KJ guided the NI DAQ implementation and data analysis activities: system identification analysis, bootstrap analysis and statistics. Together, DU-M and KJ implemented the NI DAQ system, programmed the AdeptSix 300 robotic arm, did data analysis on MATLAB, wrote the Methods section created and edited the figures. MD and FV-C provided the initial idea of giving an engineering quantitative analysis for functional benefits of the LSO balance sense in birds. They wrote most of the Discussion section and validated: (i) the design and construction of the physical model of the guinea fowl, (ii) data analysis activities, and (iii) each of the paper sections and figures. All the authors contributed to editing the paper for style, clarity, succinctness and grammar.

### Conflict of interest statement

The authors declare that the research was conducted in the absence of any commercial or financial relationships that could be construed as a potential conflict of interest.
